# The Enigmatic Roles of Caspases in Tumor Development

**DOI:** 10.3390/cancers2041952

**Published:** 2010-11-24

**Authors:** Richard Jäger, Ralf M. Zwacka

**Affiliations:** National University of Ireland, Galway, National Centre for Biomedical Engineering Science and Apoptosis Research Centre, Molecular Therapeutics Group, Galway, Ireland; E-Mail: richard.jaeger@nuigalway.ie (R.J.)

**Keywords:** caspase, cancer, apoptosis, tumor suppressor, non-apoptotic functions, mutations, LOH

## Abstract

One function ascribed to apoptosis is the suicidal destruction of potentially harmful cells, such as cancerous cells. Hence, their growth depends on evasion of apoptosis, which is considered as one of the hallmarks of cancer. Apoptosis is ultimately carried out by the sequential activation of initiator and executioner caspases, which constitute a family of intracellular proteases involved in dismantling the cell in an ordered fashion. In cancer, therefore, one would anticipate caspases to be frequently rendered inactive, either by gene silencing or by somatic mutations. From clinical data, however, there is little evidence that caspase genes are impaired in cancer. Executioner caspases have only rarely been found mutated or silenced, and also initiator caspases are only affected in particular types of cancer. There is experimental evidence from transgenic mice that certain initiator caspases, such as caspase-8 and -2, might act as tumor suppressors. Loss of the initiator caspase of the intrinsic apoptotic pathway, caspase-9, however, did not promote cellular transformation. These data seem to question a general tumor-suppressive role of caspases. We discuss several possible ways how tumor cells might evade the need for alterations of caspase genes. First, alternative splicing in tumor cells might generate caspase variants that counteract apoptosis. Second, in tumor cells caspases might be kept in check by cellular caspase inhibitors such as c-FLIP or XIAP. Third, pathways upstream of caspase activation might be disrupted in tumor cells. Finally, caspase-independent cell death mechanisms might abrogate the selection pressure for caspase inactivation during tumor development. These scenarios, however, are hardly compatible with the considerable frequency of spontaneous apoptosis occurring in several cancer types. Therefore, alternative concepts might come into play, such as compensatory proliferation. Herein, apoptosis and/or non-apoptotic functions of caspases may even promote tumor development. Moreover, experimental evidence suggests that caspases might play non-apoptotic roles in processes that are crucial for tumorigenesis, such as cell proliferation, migration, or invasion. We thus propose a model wherein caspases are preserved in tumor cells due to their functional contributions to development and progression of tumors.

## 1. Structure and Activation of Caspases

Caspases are the proteases responsible for dismantling the cell in an ordered and histologically distinct process termed apoptosis [1]. By cleaving critical proteins, caspases lead to the changes that characterize apoptosis both morphologically and biochemically, such as chromatin condensation, loss of cell adhesion, cell shrinkage, membrane blebbing, DNA fragmentation, and finally formation of apoptotic bodies, which stimulate their own engulfment by phagocytes. Caspases are expressed as inactive zymogens, the procaspases. Activation of caspases involves cleavage of their proforms into a large and a small subunit. Heterotetramers consisting of two large and two small subunits represent the active enzymes. Based on protein structure and activation mechanism, caspases can be subdivided into initiator caspases and executioner caspases. The former harbor protein-protein interaction domains at their aminoterminus, either death effector domains (DED) or caspase recruitment domains (CARD). These serve for their recruitment to specific activator platforms, where they become activated by induced proximity. The latter are substrates of initiator caspases which cleave and thereby activate them.

There are four initiator caspases, Caspase-8 and Caspase-10, Caspase-9, and Caspase-2. Upon apoptotic stimuli their proforms are recruited to specific activator platforms. Procaspase-8 (and the related Procaspase-10) is recruited to the death-inducing silencing complex (DISC) formed at the cytoplasmic portion of death receptors upon their stimulation with ligands, such as FasL or TRAIL [2,3]. Caspase-9 is activated in a large protein complex termed the apoptosome. The apoptosome forms when cellular stress signals trigger the release of cytochrome c from mitochondria. Cytochrome c induces a conformational change in the adaptor protein Apaf-1 causing it to oligomerize and recruit procaspase‑9 [4]. Caspase-2 is activated upon genotoxic stress in a large protein complex termed the PIDDosome [5].

The subgroup of executioner caspases consists of Caspase-3, -6, and -7 [1]. Caspases-1, -4, -5, and -12 are structurally similar to initiator caspases and play a role in inflammation, but not in apoptosis [6]. Caspase-14 shares the structure with executioner caspases and appears to be involved in skin differentiation [7].

## 2. Is loss of Caspase Activity Involved in Cancer?

Tumorigenesis is a multistep process wherein cells and their clonal descendents become continuously selected for acquired somatic mutations, which will allow for unrestrained growth [8]. Apoptosis represents one such potential restraint, and therefore, cancer cells are generally considered as resistant against apoptosis [8]. Do we, therefore, have to consider the components of the apoptotic machinery as tumor suppressors? If so, somatic mutations and loss of heterozygosity (LOH) in caspase genes should frequently occur in cancer. In experimental approaches, inactivation of caspases achieved by specific inhibitors or RNAi-mediated knock-down or gene knock-out should facilitate cellular transformation.

### 2.1. Somatic Mutations of Caspase Genes in Human Cancer

Ever since caspases were recognized as the fundamental proteases involved in apoptosis their expression and possible mutation in human cancer has been considered due to a potential impact on tumorigenesis and therapy resistance. A recent review article has tried to summarize these data and included information on allelic gene variants which, however, might affect tumorigenesis in an indirect manner, *i.e.*, by influencing non-tumor cells [9]. Here, we present an independent synopsis of caspases in cancer which specifically focuses on somatic mutations of caspase genes within tumor cells which may have arisen during their clonal evolution, but not due to selection pressure of cytotoxic therapy regimens. Such mutations may represent passenger alterations that are not involved in tumorigenesis but result from the general genetic instability of tumor cells [8]. Therefore, the functional impact of the mutations is of importance, as well as the occurence of LOH. Moreover, we include information about loss of expression of certain caspases which might be due to gene silencing. These data are summarized in Table 1.

#### 2.1.1. Executioner caspases

Somatic caspase-3 mutations have been detected at very low frequencies in certain human cancers, such as colon and stomach cancer, Non-Hodgkin lymphoma (NHL), and hepatocellular carcinoma [10]. In other common cancer types, such as breast and prostate cancer, mutations were absent [10,11] although caspase-3 is known to be expressed [12,13,14,15,16,17,18]. However, whether these mutations render caspases deficient in executing apoptosis was not tested.

Likewise, caspase-7 was found only rarely mutated in human cancer [19,20]. In one study tumor‑derived mutants were shown to be less capable of executing apoptosis than wildtype caspase-7 when transfected into 293T cells [19]. However, no LOH was found for the mutants, and it is unclear whether they can interfere in a dominant manner with wildtype caspase-7.

As caspase-6 mutations also occur rarely in human cancer [21,22], it can be summarized that executioner caspases are generally not mutated in the majority of human cancers. There is also not much evidence of gene silencing in tumors. Caspase-7 was found to be expressed at lower levels in the majority of colon cancers, as compared to normal mucosa [23]. Likewise, reduced expression of caspase-6 and -7 has been reported in gastric carcinoma whereas caspase-3 is expressed at higher levels than in normal mucosa [24,25]. Elevated caspase-3 levels were also measured in acute myelogenous leukemia (AML), as compared to normal peripheral blood lymphocytes [26]. Caspase-6 was found expressed in the majority of invasive carcinomas of the breast [16]. As mentioned above, caspase-3 is expressed in breast and prostate carcinoma, and expression increases with tumor progression [12,13,16]. There is only one contradictory study claiming lack of caspase-3 mRNA expression in most breast tumor samples analyzed. In benign tissue from the vicinity of the caspase-3-negative tumors, however, detection of caspase-3 transcripts also failed, raising concern whether insufficient mRNA preservation might have been the cause [27]. In conclusion, silencing of particular executioner caspases might at best be involved in a small fraction of cancers.

**Table 1:** Mutations and silencing of caspases in cancer. The percentages of cancer cases with non-silent mutations (missensse, nonsense or frameshift) or lack of expression ("silenced") of caspases are listed, respectively. Total numbers of cases examined are in brackets. Where available, information on number of tumor samples with loss of heterozygosity (LOH) or heterozygosity (het) of the mutant alleles is provided (NI: not informative), as well as information of the impact of the mutations on caspase function.

Caspase	Cancer type	mutated	silenced	LOH	Function	Reference
*Executioner caspases*						
Caspase-3	Colon	2.1% (98)				10
	NHL	0.8% (129)				10
	Lung	1.7% (181)				10
	Gastric		5% (60)			24
Caspase-6	Colorectal	2% (100)				21
	Gastric		48% (120)			25
Caspase-7	Esophagal	1% (50)			impaired	19
	Gastric		67% (120)			25
	Colon		85% (26)			23
*Initiator caspases*						
Caspase-2	Gastric		65% (120)			25
Caspase-8	Gastric	8% (162)		1 LOH, 6 het, 6 NI	impaired	44
	Liver	13% (69)			impaired	42
	Colon	5.1% (98)			impaired	43
	Neuroblastoma		75% (140)			53
	Medulloblastoma		52% (27)			47
	SCLC		79% (34)			45
Caspase-9	Colon		46% (26)			23
	Gastric		10% (60)			24
Caspase-10	Colon	2.1% (47)				37
	Gastric	3% (99)		2 LOH	impaired	41
	NHL	14.5% (117)			impaired	38
	Gastric		5% (60)			24

#### 2.1.2. Initiator caspases

If executioner caspases are expressed and functional in the majority of cancers, it appears conceivable that the initiator caspases might be functionally compromised in human cancer either by somatic mutation or by gene silencing.

In discordance with this prediction, the gene encoding the initiator caspase of the intrinsic pathway, caspase-9 is neither mutated nor silenced in the majority of cancers [11,24,28,29]. Decreased expression levels (compared to normal mucosa) have been reported in 46% of colon cancers [23], whereas in gastric cancer expression is increased [24]. While in neuroblastoma no mutations were detected [29,30], somatic mutations occurring at very low frequency in colorectal, gastric and lung carcinomas do not result in an altered amino acid sequence [28]. Thus caspase-9 does not seem to restrain tumorigenesis. It should however be noted, that in a study investigating gastric carcinomas, in 55% of cases caspase-9 was phosphorylated at Thr125 presumably preventing the activation of the enzyme [31,32].

Only little information is available about caspase-2 mutations in cancer. Caspase-2 maps at the genomic locus 7q34-35, which is frequently affected in hematological malignancies [33]. While caspase-2 expression has been shown in acute myelogenous leukemia (AML) and acute lymphoblastic leukemia [26,34], its expression is frequently lost in gastric cancer in contrast to normal mucosa [25]. No caspase-2 mutations have been detected in GIST and prostate cancer [11,35].

Caspase-8 and -10 genes are located at the genomic locus 2q33-34, a region frequently affected in cancer [36]. Caspase-10 was not mutated in breast cancer, lung cancer and hepatocellular carcinoma, but in 4.3% of colon cancer samples analyzed [37]. In 14.5% of NHL cases caspase-10 mutations have been detected that turned out to encode proteins defective in apoptosis induction when transfected into 293T cells [38]. In contrast, caspase-8 was not mutated in NHL [20], and a different study found it to be highly expressed [39]. It is, therefore, not clear whether the Caspase-10 mutants would be able to compete with wildtype caspase-8 to impair apoptosis induced by death receptors. Caspase-10 mutations are rare in acute leukemia or multiple myeloma [40]. Caspase-10 was found mutated occasionally in gastric carcinoma, with 15% LOH (in contrast to 28% LOH for mutated caspase-8 alleles), and the mutants were shown to be defective in apoptosis induction [41]. In a different study, however, the majority of gastric cancers displayed enhanced expression of caspase-10 when compared to normal mucosa [24]. Therefore, impairment of caspase-10 function is not very likely to be of relevance for gastric cancer.

A wealth of data has been published concerning caspase-8 as potential tumor suppressor. Whereas in NHL, breast and non-small cell lung cancer no mutations could be detected, a frame shift mutation was detected in 13% of hepatocellular carcinoma samples and shown to encode a caspase defective in inducing apoptosis [42]. In colon cancer, caspase-8 mutants were exclusively detected in carcinomas (5.1%), and three of the five mutants were shown to interfere with apoptosis induced by death receptor overexpression suggesting they might behave in a dominant-negative manner in the tumor cells [43]. In gastric cancer, 8% of samples contained caspase-8 mutants, remarkably all of them in advanced carcinomas [44]. These mutants were different from the colon cancer-derived ones, and most of them encoded caspase-8 molecules defective in apoptosis induction. There was no LOH in most cases, and it was not tested whether the mutants would dominantly interfere with the activity of remaining non-mutated caspase-8 (or -10) molecules.

Loss of caspase-8 gene expression by gene silencing has been reported to occur in neuroblastoma, medulloblastoma, and small cell lung carcinoma (SCLC) [45,46,47]. Caspase-8 has therefore been suggested as tumor suppressor in the neuroectodermal or neuroendocrine cell lineage, respectively [48,49]. Silencing of caspase-8 in SCLC occurs likely through DNA methylation (52% of cases) [50]. A high fraction of pediatric tumors displayed methylation of the caspase-8 gene, potentially leading to gene silencing [51]. For instance, the caspase-8 gene was found methylated in 81% of medulloblastomas analyzed [51]. In a different study, however, Caspase-8 protein was absent in only 16% of tumors, suggesting that promoter methylation might not always correlate with gene silencing [52]. Conversely, in neuroblastoma caspase-8 gene methylation correlated with amplification of the MYCN gene, whereas caspase-8 protein was absent in the majority of tumors irrespective of MYCN amplification [49,53]. In none of the cases, however, it was shown whether the silencing or loss of caspase-8 expression would occur during tumorigenesis as a result of a clonal selection process, or whether the caspase-8 gene is already silenced/methylated in the precursor cells from which the tumors originate. Of note, neural stem or progenitor cells were recently shown to lack caspase-8 expression [54]. Thus, tumors may derive from transformation of a caspase-8-negative progenitor cell.

In experiments using chicken embryos, loss of caspase-8 did not enhance the growth of tumors formed by xenotransplanted human neuroblastoma cell lines, however, was essential for their invasion and metastasis formation [55]. Caspase-8 sensitized the neuroblastoma cells to apoptosis specifically once they invaded the stroma but not within the primary tumor. Caspase-8 was therefore suggested to represent a metastasis suppressor [55]. Thus, absence of caspase-8 may become important at later tumor stages and may not be critical for growth of the primary tumor. These data are, however, not consistent with experiments that revealed functional roles of caspase-8 in migration and invasion (see below).

In summary, only a surprisingly small proportion of human cancers display inactivating mutations or silencing of caspase genes (Table 1) and it is not clear whether these events are in fact the drivers of tumorigenesis or only passengers. Moreover, some of the most prevalent cancer types, such as breast and prostate cancer, did not exhibit mutant caspase genes at all and showed expression of caspases.

### 2.2. Evidence from Transgenic Mice

How about experimental data? The most rigorous test of gene function *in vivo* is provided by transgenic mice. Using gene targeting, initiator caspase genes and several of the executioner caspase genes have been inactivated in mice [56]. A major caveat of these approaches has been embryonic lethality of caspase gene disruption (*i.e.*, caspase-3, -8 and -9) precluding assessment of tumorigenesis in adult mice. Conditional mutants which would circumvent this difficulty have so far only been published for caspase-8 [57,58]. However, no tumor-related experiments have yet been carried out with these mice.

To circumvent the perinatal lethality of caspase-9 deficient mice, fetal liver cells of these mice were used to reconstitute the immune system of lethally irradiated recipient mice [59]. Before, an Eµ-c-myc transgene had been introduced by crossing caspase-9 +/− mice with Eµ-myc transgenic mice. By this means, the impact of loss of both caspase-9 alleles on c-myc-induced lymphoma development could be measured *in vivo*. Against expectation, loss of caspase-9 did not influence lymphoma development and the severity of disease. Similar findings were obtained with Apaf-1 knock-out mice [59]. Moreover, deficiency in Apaf-1 did not enhance the rate of *in vitro* transformation of embryonic fibroblasts, which was in contrast to a previous study [60].

Also, the knock-out of the caspase-8 gene is embryonic lethal in mice. Therefore, the influence of caspase-8 deficiency on cellular transformation was tested *in vitro* using fibroblasts derived from caspase-8-deficient mouse embryos [61]. In these experiments, loss of caspase-8 reduced the number of passages required for SV40Tag-induced transformation, as assessed by soft-agar colony formation or tumor formation of cells injected into immune-deficient mice. Interestingly, caspase-8-deficient cells did not show enhanced cellular survival in soft-agar culture, indicating that the promotion of transformation was not due to an impairment of apoptosis.

In contrast to Caspases-9 and -8, disruption of the caspase-2 gene *in vivo* was not lethal and evoked only a very subtle phenotype. When caspase-2-deficient Eµ-myc compound mice were generated, lymphoma development turned out to be accelerated [62]. Interestingly, deficiency in only one caspase-2 allele was sufficient to promote lymphoma development indicating that caspase-2 might be a haploinsufficient tumor suppressor gene. Caspase-2-deficient embryonic fibroblasts could be readily transformed *in vitro* and displayed impaired apoptosis and abnormal cycling following DNA damage, implying that the DNA damage response (DRR) might represent an important tumor-suppressive mechanism involving Caspase-2 activation [62]. In this regard it is noteworthy, that tumor cells have been suggested to retain DDR-induced senescence pathways but to be deficient in DDR-induced apoptosis [63]. In mammary tumors of transgenic mice overexpressing ErbB-2 or PyMT, however, a DDR was not detectable [63], and it would therefore be interesting to assess the influence of caspase-2 loss in these tumor models.

There has only been one study published so far assessing the role of executioner caspases in tumorigenesis [64]. This study took advantage of the fact that caspase-3 gene disruption is not lethal in mice of the C57BL/6 background. Caspase-3 gene disruption was combined with an inducible c-myc transgene expressed in beta cells of the pancreas. In previous experiments it had been shown that blocking myc-induced apoptosis using a bcl-xL transgene leads to the emergence of beta cell tumors in these mice [65]. Likewise, loss of caspase-3 suppressed the myc-induced apoptosis. Against expectation, however, caspase-3 deficiency did not lead to tumorigenesis. Interestingly, loss of caspase-3 led to an inhibition of the cell cycle otherwise stimulated by sustained c-myc expression, suggesting a role of Caspase-3 in proliferation [64]. In these experiments, therefore, caspase-3 did not behave as a tumor-suppressor. It remains to be tested whether Caspase-3 activity might even be required for myc/bcl-xL-mediated tumorigenesis.

### 2.3. In Vitro Transformation of Human Cells

*In vitro* transformation of normal cells to cancer cells capable of anchorage-independent growth and tumor formation *in vivo* can be used to identify genetic or epigenetic changes that contribute to tumor formation [66]. In rodent cells two defined genetic alterations can be sufficient [67], and it has been suggested that one alteration must promote cellular proliferation whereas the second one must shut off processes detrimental to cell growth, such as senescence or apoptosis [68]. As already mentioned, deficiency in caspase-8 enhanced the rate of transformation when fibroblasts were transformed with the large T-antigen of SV40 virus. Likewise, deficiency in caspase-2 facilitated the transformation of fibroblasts by the Adenovirus E1A gene combined with active ras.

While these experiments point towards an involvement of particular caspases in tumor suppression, similar experiments using normal fibroblasts or epithelial cells of humans have never been published. It is known that human cells require five to six genetic alterations to become transformed [67], and a set of defined genetic elements has been established that is capable of transforming human cells [8,66]. However, none of these genetic elements does disrupt apoptosis as its sole function. For instance, disruption of the tumor suppressor PTEN leads to enhanced signaling via the PI3K/AKT pathway, however, this pathway not only influences cell survival but also controls cell metabolism [69]. Likewise, disruption of p53 signaling will not only affect apoptosis but also genomic stability and senescence [70]. Furthermore, apoptosis can occur independently of p53 [71]. Therefore, to date these experiments do not allow drawing conclusions about apoptosis as a mechanism of tumor suppression or caspases as tumor suppressors in human cells. This could be accomplished by introducing viral caspase inhibitors or caspase-specific shRNA constructs into cells in conjunction with other defined genetic alterations.

## 3. Why Caspase Genes Possibly Need Not be Altered in Tumors?

From clinical as well as from the limited experimental data it seems likely that loss of caspase expression or mutation of caspase genes is not a prerequisite of tumorigenesis, and that caspase genes are not classical tumor suppressor genes. There are several scenarios conceivable which may explain this counterintuitive conclusion: (i) Alternative splicing may generate dominant-negative caspase variants; (ii) caspases are blocked by inhibitory proteins; (iii) caspase-activating pathways are abrogated; (iv) caspase-independent cell death (CICD) mechanisms might circumvent the selection against functional caspase genes during tumorigenesis.

### 3.1. Scenario 1: Dominant-Negative Splice Variants of Caspases

For several caspases, alternatively spliced variants have been described, and it is conceivable that their expression is increased in tumors due to a generally deregulated splicing [72]. Shorter splice variants of caspases-2, -9, and -10 have been detected which are catalytically inactive and may interfere with apoptosis [73,74,75,76]. However, to date no study has been published addressing their expression in tumors at the transcript or protein level.

A shorter splice variant of caspase-3, termed caspase-3s, has been cloned from a human colon carcinoma cell line [77]. This variant lacks part of the carboxyterminus including the catalytic cysteine and can interfere with apoptosis in a dominant fashion. Caspase-3s was detected at transcript level in several human tissues and at protein level in human tumor cell lines [77]. The presence of this anti-apoptotic splice variant was suggested to help breast tumors tolerate high expression levels of wildtype caspase-3 [78]. However, differential expression was only assessed at the mRNA level, and it is not clear whether caspase-3s protein amounts would indeed be sufficient to counteract caspase-3 activity.

A longer transcript of caspase-8 generated by alternative splicing has been termed caspase-8L [79]. Due to a frameshift this longer mRNA encodes a truncated protein lacking the carboxyterminal region including the catalytic site. caspase-8L was shown to dominantly interfere with apoptosis by preventing the binding of caspase-8 to the DISC [80]. It was detected by western blotting in AML and ALL and suggested to protect their progenitors from regulatory apoptosis [81]. In a small panel of neuroblastomas, caspase-8L transcripts could be detected in six out of six undifferentiated tumors, but only in two out of five differentiated tumors [82]. Because most anti-caspase-8 antibodies will not discriminate between caspase-8 and -8L, it is currently unclear to which extent caspase-8L may have contributed to the caspase-8 expression detected in the immunohistochemical analyses of various cancer types.

### 3.2. Scenario 2: Endogeneous Inhibitors of Apoptosis Block Caspases in Tumor Cells

There are two major classes of endogeneous caspase inhibitors. One class is represented by the Flice inhibitory protein (c-FLIP) (and its variants) which shares homology with Caspase-8 and -10 but is enzymatically inactive [83]. Via its DED domain it can compete with Caspase-8 (-10) for binding to the DISC, hence preventing activation of these caspases. c-FLIP was found overexpressed in many human tumor types, such as prostate carcinoma (96.3%), hepatocellular carcinoma (83.7%), colorectal (68.8%) and gastric cancer (57.1%) and over 90% of Hodgkin lymphomas [84,85,86,87,88]. In other tumor entities, such as T-Lymphoma or Burkitt lymphoma, c-FLIP is absent [87], and in gastric cancer c-FLIP overexpression is not inversely correlated with the percentage of apoptotic cells, implying non-apoptotic functions [86]. c-FLIP and caspase-8 signaling have recently been shown to play a role in the proliferation of B-lymphocytes [89,90] and in invasive processes of tumor cells [91,92], and stimulation of these processes might also confer a selective advantage to tumor cells.

The other class comprises inhibitor of apoptosis proteins (IAPs) which share in common at least one baculovirus IAP repeat (BIR) domain [93]. Although initially thought of preventing apoptosis by binding to and blocking caspases, it turned out that only one member, the X-linked inhibitor of apoptosis protein (XIAP), is actually capable of doing so [93]. Therefore, in the context of caspases in cancer, we will only focus on XIAP and not discuss potential roles of the other IAPs which have been described to be overexpressed in various cancer types [94]. XIAP contains three BIR domains (BIR1-3) and a C-terminal RING domain. BIR-2 and -3 bind caspases-3/-7 or caspase-9, respectively [95]. The RING domain works as E3 ubiquitin ligase mediating ubiquitination of target proteins [96].

XIAP has been shown to be overexpressed in several types of human cancer [97,98,99,100,101,102,103,104,105]. Thus, it is possible, that XIAP-mediated inactivation of caspases-3, -7, and -9 would spare the selection for mutations in (or silencing of) these caspases during tumorigenesis. In breast cancer, however, activated caspase-3 and presence of caspase-activated DNAse was observed despite of XIAP expression [106]. Also in colorectal carcinomas as well as in non-small cell lung carcinoma (NSCLC) and in hepatocellular carcinoma there was no inverse relationship between XIAP expression and percentage of apoptotic cells [98,100]. These observations suggest that the XIAP expression observed in tumors does not suffice to prevent apoptosis or caspase activation. Moreover, as XIAP blocks only particular caspases, the remaining caspases might still be active, therefore necessitating other mechanisms of their inactivation if this should be required at all.

XIAP (and other IAPs) are bound and counteracted by certain other proteins such as Smac/DIABLO or XAF1 [107,108,109]. Smac becomes released from mitochondria upon apoptotic stimuli [108]. Smac expression is reduced or lost in several tumor entities [110]. However, a considerable fraction of each of these tumor entities still displays Smac expression. Of note, none of the studies addressed the subcellular localization of Smac. If retained inside mitochondria, Smac would not impair cytosolic XIAP function. Likewise, reduced XAF1 expression has been described in several human cancers, and XAF1 has been suggested to represent a tumorsuppressor [111,112,113,114,115,116]. Interestingly, in gastric carcinomas the XIAP/Smac mRNA expression ratio was reported to be similar to normal tissue, whereas the XIAP/XAF1 ratio was significantly increased [117]. Therefore, the loss of specific inhibitory proteins could free XIAP to promote tumorigenesis.

In this regard, a growing body of evidence suggests that the upregulation of XIAP in tumors might reflect functions that are unrelated to caspases. It has been shown that XIAP can ubiquitinate non-caspase proteins which are only indirectly affecting apoptosis [118]. Among them is for instance the tumor suppressor PTEN [119]. Thus, upregulating XIAP could confer a selective advantage to tumor cells independent of caspase inhibition.

Experiments using XIAP knockout mice yielded conflicting results. Specific deletion of the RING domain of XIAP improved the survival of lymphoma-prone Eµ-myc transgenic mice, possibly by enhancing apoptosis [120]. On the other hand, XIAP deficiency did not prevent the emergence of prostate tumors in the transgenic TRAMP model, but rather led to a faster disease progression [121]. Both of these experiments did not address non-caspase targets of XIAP. Interestingly, another study revealed that XIAP may contribute to metastasis formation by interacting with the IAP family member Survivin, thereby leading to Nuclear Factor Kappa B (NF-κB) -mediated upregulation of integrins [122]. This is an interesting parallel to the putative role caspase-8 plays in invasion and metastasis of neuroblastoma cells [55]. These findings indicate that proteins previously thought to control apoptosis might be of little importance during early tumorigenesis but play major non-apoptotic roles during progression towards metastasis.

### 3.3. Scenario 3: Pathways Upstream of Caspase Activation are Disrupted

The simplest explanation why in tumors caspases need not be mutated or silenced is that pathways upstream of their activation are disrupted. One possibility is the absence of death receptor signaling, which might be due to loss of expression, as shown for example for TRAIL receptors in tumors [123]. TRAIL receptor activity can be modulated by highly homologous decoy receptors which, however, do not contain the functional intracellular domains required for DISC formation. Therefore, a high level of decoy receptor expression may also prevent the TRAIL ligand from activating caspases [124]. Furthermore, death receptor signaling may be blocked intracellularly by FLIP or by dominant-negative caspase-8 variants (see previous chapters). On the other hand, signaling pathways, such as PI3K/Akt or the Ras/MAPK pathway, often are hyperactivated in cancer cells and may prevent apoptosis by various means, *i.e.*, by phosphorylation of Bcl-2 family proteins [125,126] or of caspase-9 [32]. Moreover, mutations in or loss of p53 may compromise apoptosis [127]. Recently, it was suggested that tumor cells are characterized by sustained DDR signaling that, however, the branch leading to apoptosis (via p53) is specifically abrogated, whereas senescence signaling is still retained [63]. Contradicting this notion, there was no DDR signaling detectable in classical transgenic breast cancer models [63]. Also in human cancer, markers of DDR signaling tend to be absent at later progression stages [128,129].

Given the considerable fraction of apoptotic cells observed in several human cancer types (see below), tumor cells are generally capable of undergoing apoptosis and of activating caspases. Likewise, in several transgenic tumor models tumorigenesis occurs despite of high frequencies of apoptosis within normal epithelium and tumors [130,131]. The molecular pathways governing this spontaneous apoptosis have not yet been identified; however it is evident that they must be capable of overriding the survival pathways hyperactivated in tumor cells.

### 3.4. Scenario 4: CICD Circumvents the Need for Caspase Dysfunction

A further explanation for the infrequent mutation or silencing of caspase genes in tumors might be the occurrence of caspase-independent cell death (CICD) [132]. If cells destined to die will do so irrespective of caspase activity, then abrogation of caspase function will not provide a selective advantage.

CICD has been demonstrated by treating cultured tumor cells with chemotherapeutic drugs in the presence of caspase inhibitors and assessment of either cell viability or clonogenic survival ofcells [133,134]. When AML blasts from patient bone marrow were treated with chemotherapeutic drugs, caspase inhibition did not protect them from cell death [133]. Similarly, a pan-caspase inhibitor was unable to restore clonogenic growth of HeLa cells exposed to cytotoxic drugs [134]. In both experiments CICD was associated with loss of mitochondrial membrane potential. The latter also occurs during a distinct form of CICD termed necroptosis (or programmed necrosis), which is observed when death receptors are stimulated but caspase activation is blocked [135]. Recently, a complex formed between the receptor-interacting serine-/threonine protein kinases RIP1 and RIP3 was shown to participate in the decision to either undergo apoptosis or necroptosis [136].

Besides a potential role in tumor therapy, there is so far no evidence of CICD taking place during tumorigenesis. This may be due to the difficulty to readily identify CICD on histological sections. There is some experimental evidence that CICD might occur in c-myc-induced mammary tumors of transgenic mice, because dying cells in such tumors displayed non-apoptotic morphology and tumors lacked caspase-3 activity [137]. As dying tumor cells were TUNEL-positive, the possibility remains that also in human tumors TUNEL-positive cells may actually die in a caspase-independent manner. In most of the respective publications, however, apoptosis was assessed based on morphological criteria in addition to TUNEL.

## 4. The Occurrence of Spontaneous Apoptosis in Tumors

As mentioned, the scenarios delineated above are difficult to reconcile with the significant spontaneous apoptosis rates observed in several human tumor types which often exceed those of corresponding normal tissues [138,139,140,141,142,143,144,145,146,147,148,149]. In fact, spontaneous apoptosis occurring in malignant neoplasms was already noted in the seminal publication of Kerr *et al.* (1972) that coined the term apoptosis [150]. The assessment of apoptosis in tumors, however, is not free of potential errors [151]. These might result from different sensitivities of the methodologies used, such as morphological assessment or detection of DNA fragmentation by the TUNEL or ISEL method. Likewise, the duration of apoptosis and of clearance of apoptotic bodies may vary between benign and transformed tissue. In addition, the time that has passed between resection and fixation of tumor material may influence the degree of DNA fragmentation. Despite of all these possible variables, the presence of apoptotic cells within tumors has undoubtedly been established, in most cases based both on detection of DNA fragmentation and on morphological criteria.

It has been argued that in some cases the apoptotic cells identified within tumors may actually represent infiltrating lymphocytes which are undergoing apoptosis [152]. In breast cancer, however, lymphocyte infiltration and rate of apoptosis did not show correlation [153]. In addition, tumors in certain murine transgenic cancer models contain a high number of apoptotic cells which can be decreased by overexpressing anti-apoptotic transgenes specifically in tumor cells [130,131]. Therefore, it is unlikely that the apoptosis observed in tumors is primarily due to infiltrating lymphocytes.

The proportion of apoptotic cells in tumors is commonly expressed as the apoptotic index (AI). For small cell lung carcinoma an AI of 2.65% (mean) was reported [147], for prostate cancer an AI of 1.9% [148], and for gastric cancer an AI of 1.97% [146]. Several studies on breast cancer have even demonstrated a correlation between the rate of apoptosis and tumor progression [138,139,142]. In one study [138], the AI in breast cancer varied from 0.02% for hyperplasia up to 3.69% for invasive carcinoma (median 1.17%). Two independent studies on colon cancer found the AI to increase from hyperplasia to adenoma and then to decline again in carcinoma, however to values still higher than measured in benign tissue [141,143]. The AI increased also during progression of endometrial cancer, with an AI of 1.17% for benign proliferative endometrium, AIs of 2.2% and 2.57% for simple and complex hyperplasia, respectively, and an AI of 3.31% for endometrial carcinoma [140].

These findings point towards a possible role of apoptosis in promoting tumor progression which can be envisaged in at least two ways: first, by stimulating macrophage infiltration; second, by increasing the number of cell divisions. Apoptotic cells are known to attract macrophages [154]. In normal tissues this provides for the rapid clearance of apoptotic bodies. In a transgenic breast cancer model, infiltrating macrophages have been shown to promote angiogenesis and progression of tumors [155]. From human cancer, a correlation between tumor-associated macrophages and progression is well known [156]. In cancer apoptotic cells may thus constitute a source of chemo-attractants stimulating macrophage infiltration and tumor progression.

In most tumor types analyzed, the AI correlated with the percentage of proliferating cells (the mitotic or labeling index [MI]) suggestive of a high turnover of cells [138,139,142,143]. Such high turnover might directly be linked with tumor progression because the likelihood of acquiring secondary mutations increases with the number of cell divisions. Indeed, assuming the size of a tumor initially being restrained (*i.e.*, by interstitial pressure or limiting blood supply), a high rate of apoptosis would allow for more cell divisions until this critical size is reached [157]. Likewise, in an occult or dormant tumor of a constant size the continuous disappearance of cells by apoptosis would allow for a certain degree of proliferation [158]. By this means, mutations might accumulate and lead to increased malignancy of cells [159].

In both ways, the promotion of tumor progression would depend on an intact basal apoptotic machinery, *i.e.*, functional caspases. Mobilization of macrophages has been suggested to involve the caspase-dependent release of chemo-attractant factors from apoptotic cells [160]. The enhancement of cell proliferation facilitated by apoptotic tumor cells could be directly stimulated by signals emitted from the latter. In Drosophila, caspases play a role in a phenomenon termed compensatory proliferation, wherein cells undergoing apoptosis stimulate neighboring cells to enter the cell cycle [161]. Compensatory proliferation has been observed to occur within the wing and eye imaginal discs. Different mechanisms are involved in compensatory proliferation occurring in proliferating *versus* differentiating tissues. Whereas the former is dependent on the initiator caspase DRONC and involves dpp/wg signaling, the latter requires the executioner caspases DrICE and Dcp-1 which are required to activate Hh signaling [161]. Compensatory proliferation induced by apoptotic cells has also been demonstrated to play a role in head regeneration in the cnidarian Hydra [162]. In mice, and probably in humans as well, compensatory proliferation is playing a role in liver cancer [163]. The concept, that apoptotic cells emit signals that stimulate the proliferation of neighboring cells would fit well with the concurrent presence of apoptotic and proliferating cells within tumors. It is intriguing to speculate that caspase activity might promote the release of the respective pro-proliferative signaling molecules.

## 5. Non-Apoptotic Functions of Caspases Possibly Involved in Tumorigenesis

If apoptosis is indeed contributing to tumor progression, then it might not seem too surprising to find caspase genes neither mutated nor silenced in the majority of cancer types. Furthermore, if caspases are present and functional, their various non-apoptotic activities might be co-opted by tumor cells and fulfill *essential* functions during tumorigenesis.

Caspases have been shown to be involved in the differentiation of several cell types, such as monocytes, muscle precursor cells, glial cells, lens cells, and embryonic stemcells [164,165,166,167,168]. These functions would possibly counteract the tumorigenic transformation of cells which is characterized by the loss of specific differentiation markers. On the other hand, non-apoptotic functions of caspases have been described which might contribute to tumorigenesis. These comprise cellular proliferation, migration and invasion (see Table 2). Disturbances of the signaling pathways that normally govern correct cellular differentiation within the tissue context may at the same time stimulate proliferative, migratory and invasive programs that depend on non-apoptotic activities of caspases.

### 5.1. Caspases in Proliferation

There are several lines of evidence that caspases play a role in cellular proliferation. Caspase-3 expression and activity was found to periodically fluctuate in a cell cycle-dependent manner in HeLa cells, with a peak at the G2/M transition [169]. Treatment of HeLa cells with a caspase inhibitor, however, did not interfere with proliferation but rather with the mitotic check-point control [169]. Recently, also caspases-7 and -8 were shown to be periodically activated during mitosis of cultured tumor cell lines, and specific interference with caspase-7 expression led to mitotic arrest, raising the possibility that caspase-7 might be crucial for the proliferation of tumor cells *in vivo* [170]. In transgenic mice, loss of caspase-3 abrogated the c-myc-induced hyper-proliferation of pancreatic beta cells, suggesting that caspase-3 activity might be important in the cell cycle of tumor cells [64]. Using chemical inhibitors it was shown that caspase-6 is required for B-lymphocytes to enter the cell cycle following quiescence [171]. Studies using transgenic mice have demonstrated a similar role of caspase-8 in T-cells [58]. Furthermore, caspase-8 activity is involved in the cytokine-induced proliferation of hematopoietic progenitor cells [172].

### 5.2. Caspases in Migration and Invasion

Several studies have implicated caspase-8 in the migration of tumor cells. Embryonic fibroblasts derived from caspase-8 knockout mice display reduced motility, probably due to suboptimal activation of calpains [173]. In most studies using tumor cell lines, the catalytic activity of caspase-8 was dispensable for the stimulation of migration [91,174,175,176]. One study showed that particularly the DED domain of caspase-8 is required for EGF receptor-mediated cellular migration [176]. Overexpression of a catalytically inactive caspase-8 mutant was shown to confer migratory and metastastic behavior to neuroblastoma cells [175]. In contrast, catalytically active caspase-8 has been implicated in the acquirement of invasive properties in TRAIL-stimulated colon cancer cells expressing a mutant form of PI3 kinase [91]. This process required caspase-8-mediated cleavage of ROCK-1, a regulator of actin cytosceleton dynamics [91].

Two studies point towards an involvement of caspase-3 in motility and invasion. Caspase-3-mediated cleavage of phospholipase A2 has been implicated in the laminin-induced migration of ovarian carcinoma cells [177]. Catalytically active caspase-3 was also implicated in the invasive behavior of a rat hepatoma cell line under hypoxic growth conditions [178].

**Table 2:** Non-apoptotic functions of caspases which might play a role in tumorigenesis. (n.d., not determined; Ref., reference).

Function	Caspase	Experimental system	Catalytic activity required?	Ref.
**Proliferation**	Caspase-3	• mitotic check-point control of HeLa cells	yes	[169]
		• c-myc-induced hyperproliferation of pancreatic beta-cells *in vivo*	n.d.	[64]
	Caspase-7	• caspase-7 knockdown leads to mitotic arrest in HepG2 cells	n.d.	[170]
	Caspase-6	• reentry into cell cycle of quiescent B-cells	no	[171]
	Caspase-8	• reentry into cell cycle of quiescent T-cells	n.d.	[58]
		• cytokine-induced proliferation of hematopoietic progenitors	yes	[172]
**Migration**	Caspase-3	• laminin-induced migration of ovarian carcinoma cells	yes	[177]
	Caspase-8	• Caspase-8-deficient embryonic fibroblasts display reduced motility	n.d.	[173]
		• EGFR-mediated migration of neuroblastoma cells	no	[176]
		• interaction between Caspase-8 and Calpain 2 involved in migration of neuroblastoma cells	no	[175]
**Invasion**	Caspase-3	• invasive behavior of rat hepatoma cells	yes	[178]
	Caspase-8	• Caspase-8 cleaves ROCK in TRAIL- stimulated colon cancer cells	yes	[91]

## 6. Concluding Remarks

**Figure 1:**
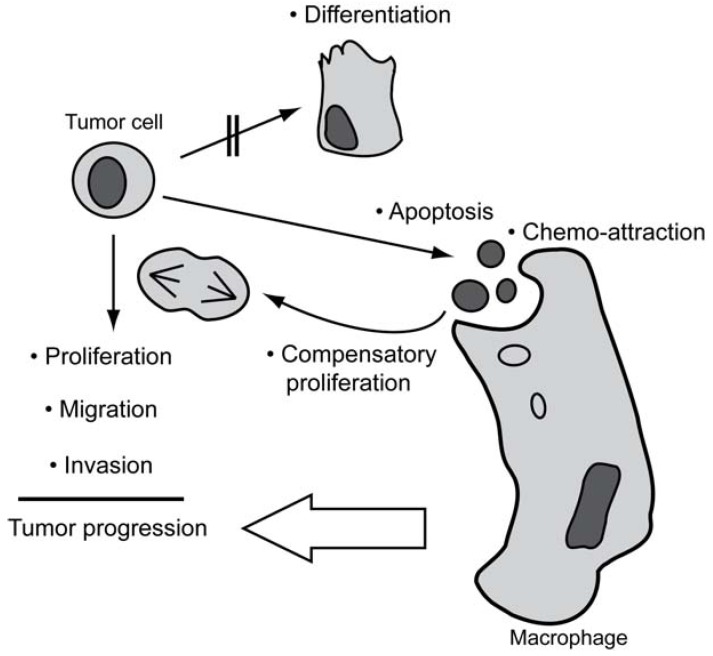
Established functions (•) of caspases and their potential contribution to tumor progression. By triggering apoptosis, caspases will provoke compensatory proliferation and chemo-attraction of macrophages. The latter may indirectly accelerate the progression of tumors. When the differentiation-inducing functions of caspases are blocked, caspases may promote proliferation and stimulate migratory behavior and invasiveness of tumor cells that are not undergoing apoptosis.

Loss of functional caspases is not a common event in human cancer, and also evasion of apoptosis does not seem to represent a general hallmark of cancer. To comply with the occurrence of apoptosis and retainment of caspases in tumors, we therefore propose a model (Figure 1) wherein tumor cells are sensitized to apoptosis as a consequence of being subjected to growth-promoting stimuli under stressful growth conditions, such as hypoxia. This results in a high rate of spontaneous apoptosis which, however, will stimulate compensatory proliferation and macrophage infiltration, thus promoting tumor progression. As a corollary, the basal apoptotic machinery has to remain functional, and surviving tumor cells can adopt the various non-apoptotic functions of caspases to help overcoming growth restrainments, allowing for further progression.

Support for this model comes from two recent studies showing that genetic ablation of a pro‑apoptotic molecule did not promote but unexpectedly impaired radiation-induced tumor development in a transgenic model of thymic lymphoma. The authors argued that apoptosis would stimulate the mobilization and compensatory proliferation of hematopoietic stem cells, allowing for expansion of mutant clones [179,180]. If this model holds true, then anticancer therapies aimed at inducing apoptosis would bear the risk of actually increasing the likelihood of further malignant progression or development of secondary cancer.

Why does our model seem at first counterintuitive and contradictory to the common opinion? So far, the importance for tumorigenesis of evading apoptosis may simply have been overstated due to the fact that acquired apoptosis resistance during therapy represents a major obstacle to cancer cure. Moreover, many of the experiments analyzing apoptotic players have been carried out using established cell lines. Adaptation to cell culture conditions may strongly select for tumor cells evading apoptosis. Finally, attempts to bolster these *in vitro* data with published clinical data may sometimes have led to a bias in favor of citations confirming loss of caspase function.

In contrast, this review highlights the degree of uncertainty that still exists with respect to caspase function in tumorigenesis. Apart from the complexity of the apoptotic machinery, a main reason for that may be the still too limited analysis of human tumor material. Only few studies have so far undertaken an analysis of the activation of particular caspases in relationship to expression of inhibitors within the same tumor specimen [106]. To adequately address the complex interplay between the various caspases and splice variants, their inhibitors, and the proteins blocking the inhibitors themselves (such as XAF1 or Smac), it will be important to study the concomitant expression and mutation status of these apoptotic players on a single cell level or at least on serial sections of the same tumor specimens. Also the transgenic *in vivo* approaches need further improvement, e.g. by conditionally disrupting caspases or by overexpressing specific or general caspase inhibitors in mouse models that closely recapitulate the process of tumorigenesis in humans.
